# Application of Levan-Rich Digestate Extract in the Production of Safe-to-Use and Functional Natural Body Wash Cosmetics

**DOI:** 10.3390/molecules27092793

**Published:** 2022-04-27

**Authors:** Tomasz Wasilewski, Artur Seweryn, Dominika Pannert, Kinga Kierul, Marta Domżał-Kędzia, Zofia Hordyjewicz-Baran, Marcin Łukaszewicz, Agnieszka Lewińska

**Affiliations:** 1 Department of Industrial Chemistry, Faculty of Chemical Engineering and Commodity Science, Kazimierz Pulaski University of Technology and Humanities in Radom, Chrobrego 27, 26-600 Radom, Poland; tomasz.wasilewski@uthrad.pl; 2 Research and Development Department, ONLYBIO.life S.A., Jakóba Hechlińskiego 6, 85-825 Bydgoszcz, Poland; dominika.pannert@boruta-zachem.pl; 3 Research and Development Department, INVENTIONBIO S.A., Jakóba Hechlińskiego 4, 85-825 Bydgoszcz, Poland; kinga.kierul@inventionbio.pl (K.K.); marta.domzal@inventionbio.pl (M.D.-K.); 4 Faculty of Biotechnology, University of Wroclaw, Joliot-Curie 14a, 50-383 Wroclaw, Polandmarcin.lukaszewicz@uwr.edu.pl (M.Ł.); 5 Lukasiewicz Research Network-Institute of Heavy Organic Synthesis “Blachownia”, Energetykow 9, 47-225 Kedzierzyn-Kozle, Poland; zofia.hordyjewicz@icso.lukasiewicz.gov.pl; 6 Faculty of Chemistry, University of Wroclaw, Joliot-Curie 14, 50-383 Wroclaw, Poland

**Keywords:** plant extracts, *Bacillus subtilis*, cosmetics, safety, skin irritation, quality

## Abstract

The study focused on the evaluation of the possibility of using a levan-rich digestate extract in the production of safe and functional body wash cosmetics. Model shower gels were designed and formulated on the basis of raw materials of natural origin. Prepared prototypes contained various extract concentrations (16.7; 33; 50%). A gel without extract was used as a reference. The samples were evaluated for their safety in use and functionality. The results showed that the use of high-concentration levan-rich digestate extract in a shower gel resulted in a significant reduction in the negative impact on the skin. For example, the zein value decreased by over 50% in relation to the preparation without the extract. An over 40% reduction in the emulsifying capacity of hydrophobic substances was also demonstrated, which reduces skin dryness after the washing process. However, the presence of the extract did not significantly affect the parameters related to functionality. Overall, it was indicated that levan-rich digestate extract can be successfully used as a valuable ingredient in natural cleansing cosmetics.

## 1. Introduction

In terms of composition, shower gels are typically formulated as aqueous solutions of anionic surfactants and additionally enriched with compounds from the groups of non-ionic and amphoteric surfactants and various additives, including preservatives, colorants, plant extracts or pH regulators. The viscosity of these formulations is usually modified by adding sodium chloride or using polymers [1,2].

In recent years, cosmetics manufacturers have been increasingly turning their interest towards the development and production of natural cosmetics. This shift has largely been driven by changing consumer expectations and the search for safe products. There have been a number of scientific studies evaluating the application of various types of plant-based raw materials [3,4] or ingredients obtained through biotechnological processes [5,6,7,8] as cosmetic components.

A key aspect in evaluating the quality of cosmetics, especially the types intended for personal hygiene, is the maximum possible reduction in adverse effects on the skin surface. It is expected that the washing process leaves the skin in the best possible condition, without any undesirable effects such as skin irritation, redness or excessive dryness. Based on the current state of knowledge, measures to reduce the skin irritating effect of body wash cosmetics rely on mechanisms that minimize interactions between cosmetic ingredients and epidermal structural proteins. These mechanisms relate to changes in the structure of surfactant aggregates forming in the wash bath which contribute to the development of skin irritation as a result of additives incorporated into the cosmetic formulation. Remedial measures consist of lowering the concentration of free surfactant monomers in the solution as they are primarily responsible for interactions with skin proteins or changes in the structure of emerging micellar aggregates [9]. These effects are achieved by adding surfactants with a documented low skin irritation potential [1,10,11], polymers [2,12], divalent metal salts [13], hydrophobic substances [14] or plant extracts [3,4] to cosmetic formulations.

A particularly interesting trend in the development of cosmetics is the application of plant-based raw materials and ingredients obtained in biotechnological processes, including fermentation [15,16].

The use of fermented ingredients in cosmetic formulations is becoming more and more popular in modern cosmetics. These types of ingredients increase the bioavailability of important ingredients with a cosmeceutical effect, making them stronger and more effective than normal. They facilitate the absorption of the cosmetic into the deeper layers of the skin. The fermentation process itself enables the synthesis of new substances that can positively affect the condition of the skin, and their natural production reduces the risk of irritation. These ingredients can also influence the skin microbiome, positively influencing the natural microflora and preventing excessive growth of pathogenic microflora. Fermented ingredients can enrich the finished cosmetic formulation with various anti-aging or beautifying compounds and visibly improve the condition of the skin. Compounds that can be identified in various types of fermented solutions include amino acids and peptides, vitamins, enzymes, minerals, antioxidants, etc. Extract of rice bran fermented with *Aspergillus oryzae* showed higher tyrosinase and elastase inhibition activity than other tested extracts [17]. Red ginseng *Panax ginseng* (Order: *Apiales*, Family: *Araliaceae*, Genus: *Panax*) fermented with *Lactobacillus brevis* showed greater skin whitening and anti-wrinkle activity than unfermented ginseng [18]. *Bacillus subtilis* is a bacterium known to synthesize various compounds for cosmetic use. One of the ingredients often found in cosmetics is Bacillus Ferment. It is used as a source of proteolytic enzymes that gently exfoliate dead cells. It can improve the penetration of various substances through the skin [19]. However, raw materials obtained by fermentation with *B. subtilis* may also show other properties depending on the conditions of the process carried out. It has been shown that *B. subtilis* natto—fermented *Radix astragali*, which is dried root of *Astragalus membranaceus* (Order: *Fabales*, Family: *Fabaceae or Leguminosae*, Genus: *Astralagus L.*)—had a stimulating effect on collagen synthesis in human fibroblast and keratinocyte cells [20] and also had a positive effect on the synthesis of hyaluronic acid [21]. *B. subtilis* is also capable of synthesizing other compounds of interest for cosmetic use, e.g., biopolymers. One of these is levan, which is a fructose polymer. Its potential in cosmetic applications has already been demonstrated [16]. The polymer itself has antioxidant properties [22], is able to increase skin hydration [23,24] and is not toxic to human cells [16]. As a polymer, it can also reduce the irritating effects of other components, in particular those of surfactants [9].

The main goal of our research was to show that fermentation ingredients are not a short-lived fashion in cosmetics but that they are an important step towards a sustainable industry, and fermentation biotechnology could become a turning point in the development of highly functional cosmetics. The aim of the presented study was to demonstrate the reduction of the irritating effect of effective ionic surfactants in the formulations of washing cosmetics with the use of ferment rich in the biopolymer levan (*Bacillus* Ferment Extract), based on the example of the developed model recipe. Verification of the aim required empirical tests, including the assessment of safety in use (zein value, bovine serum albumin test) and functionality (foaming ability, detergent properties, color, rheological characteristics and microbiological testing).

## 2. Results and Discussion

### 2.1. The Optimization of the Fermentation Process Focused on Obtaining Ferment Rich in Levan, Polymer and Divalent Metals Ions

The type and concentration of inorganic salt can be translated into the structure of the bulk phase and the performance properties. Our previous research [25] showed that magnesium salt resulted in more favorable characteristics than sodium salt due to the surface activity of the formulations. Additionally, the formulations in which magnesium salt was used caused much less irritation compared with the formulations with monovalent ions. The ICP analysis allowed the determination of the content of monovalent and divalent ions in the obtained *Bacillus*-fermented supernatant. A sample of 50 g of tested fermented material contained 4.14 mg of magnesium, 69.44 mg of sodium, 96.34 mg of potassium, 2.90 of calcium, 0.078 mg of zinc, 30.83 mg of phosphorus and 8.39 mg of sulfur ions. Sodium, potassium, calcium, magnesium and phosphate are some of the components of NMF (natural moisturizing factor) [26]. These minerals are capable of restoring moisture as a result of their hygroscopic characteristics. Both calcium and magnesium ions support the induction of skin barrier repair mechanisms and improve its functions [27,28]. Calcium ions also play an important role in regulating keratinocyte differentiation [28,29]. The healing effects of sulfur waters have been known for centuries. Sulfur-containing waters have a keratolytic effect, gently exfoliating the epidermis [30]. They also show bactericidal and antifungal properties [31]. The presence of various ions is also of a utility nature. Products containing monovalent metal salts are characterized by good performance parameters, i.e., the ability to wash, foam and emulsify fat, while divalent metal salts allow to obtain products with a high degree of safety in use [13].

**Figure 1:**
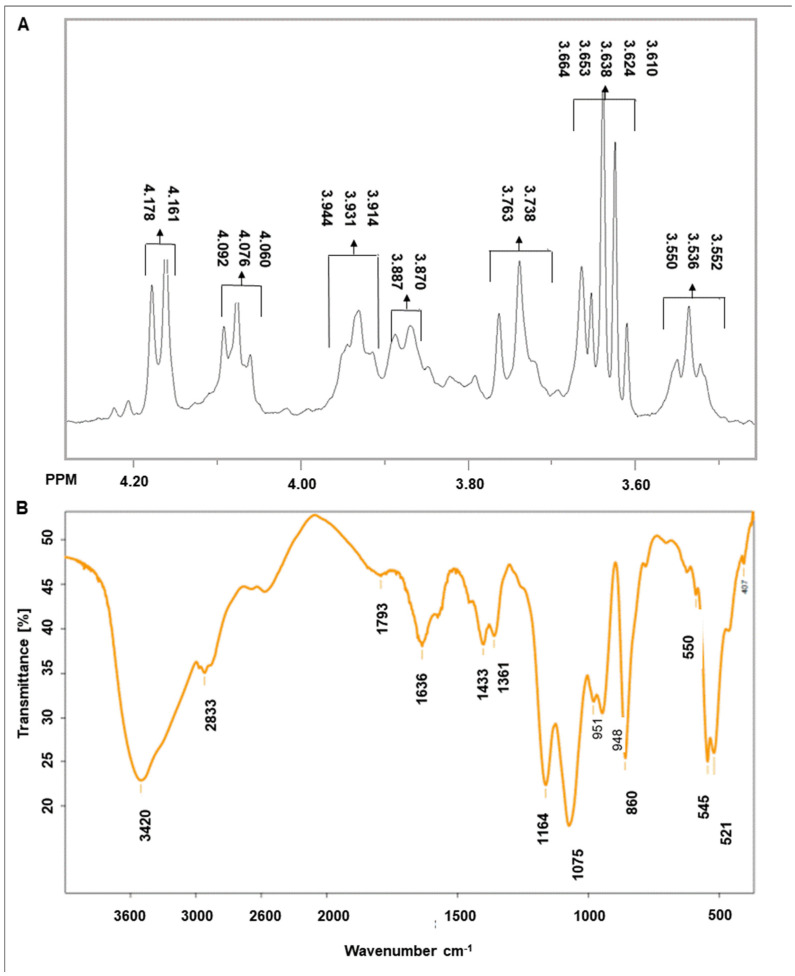
Spectra of levan from *B. subtilis* natto KB1—(**A**) ^1^H NMR, (**B**) IR.

Polymer and surfactants often appear side by side in emulsions, suspensions and other colloidal systems in many products relevant for the food, pharmaceutical and chemical industries. Mixtures of polymer and surfactant can exhibit molecular interactions that might affect the physicochemical properties of the system and result in an influence on thickening or stabilizing. The addition of a polymers to formulations containing anionic surface-active agents reduces the irritant potential of the surfactant [9]. Spectroscopic analysis confirmed the presence of levan, a fructose polymer, in the tested material obtained after fermentation of *B. subtilis* natto KB1. The obtained data are comparable with those obtained previously for this bacterial strain [16]. According to the ^1^H NMR spectrum, seven signals of chemical shift were observed at 4.17 (H-3), 4.08 (H-4), 3.93(H-5), 3.88 (H-6a), 3.75 (H-1a), 3.64 (H-1b) and 3.55 (H-6b) ppm. The FT-IR spectrum exhibited a strong band at 3430 cm^−1^, which was attributed to the hydroxyl (- OH) stretching vibrations of the polysaccharide. Bands from the carbon–hydrogen (C–H) stretching vibration were around 2933 cm^−1^, which confirmed the existence of fructose residue. The band at 1433 cm^−1^ was attributed to C–H. The bands around 1075 cm^−1^ were attributed to stretching vibrations of the glycosidic linkage C–O–C and C–OH groups. The absorption around 951 cm^−1^ was attributed to the stretching vibrations of the pyran ring. The band around 1636 cm^−1^ was evidence of bound water. The concentration of levan in the *Bacillus*-fermented supernatant was 5.04 ± 0.19%. Relevant spectra are presented in Figure 1.

### 2.2. Antimicrobial Activity of Ferment Rich in Levan

**Figure 2:**
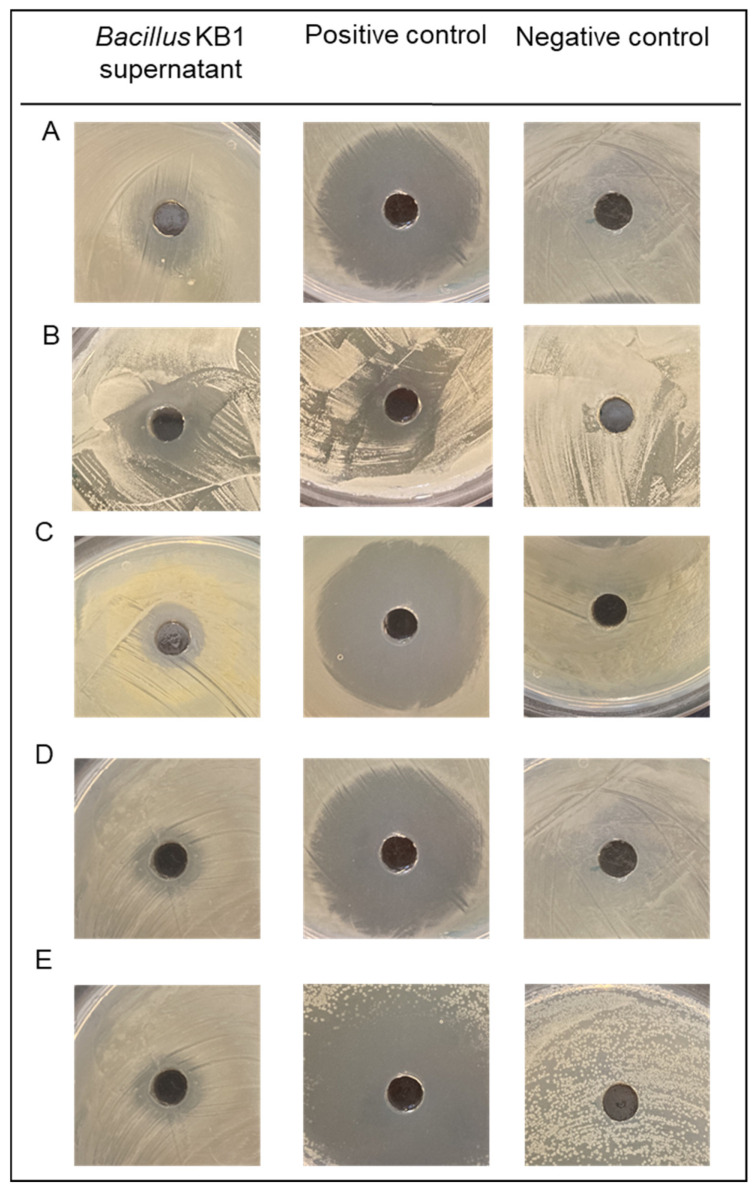
Antimicrobial activity of *Bacillus*fermented supernatant against bacterial and yeast pathogens; (**A**) *P. aeruginosa*, (**B**) *C. albicans*, (**C**) *S. aureus*, (**D**) *E. coli*, (**E**) *S. epidermidis*.

The compounds obtained through the fermentation process with the use of various species of microorganisms and their use in cosmetic products is known. Thanks to the fermentation process, compounds are formed that are often characteristic of the microorganism used, with various effects. In the case of *B. subtilis* fermentation, antimicrobial, prebiotic and other compounds, including proteolytic enzymes, are formed. The *Bacillus*-fermented supernatant was tested against pathogenic microorganisms. Its antimicrobial activity, reducing cell biofilm and preventing their adhesion to the surface, was determined. First, the antimicrobial potential (Figure 2) and the MIC values were determined (Table 1).

The antimicrobial potential was initially assessed in the agar diffusion test. After incubating the pathogenic microorganisms with the *Bacillus*-fermented supernatant, a visible zone of inhibition was observed (Figure 2). In the further course of the study, the MIC value of each test microorganism was determined.

**Figure 3:**
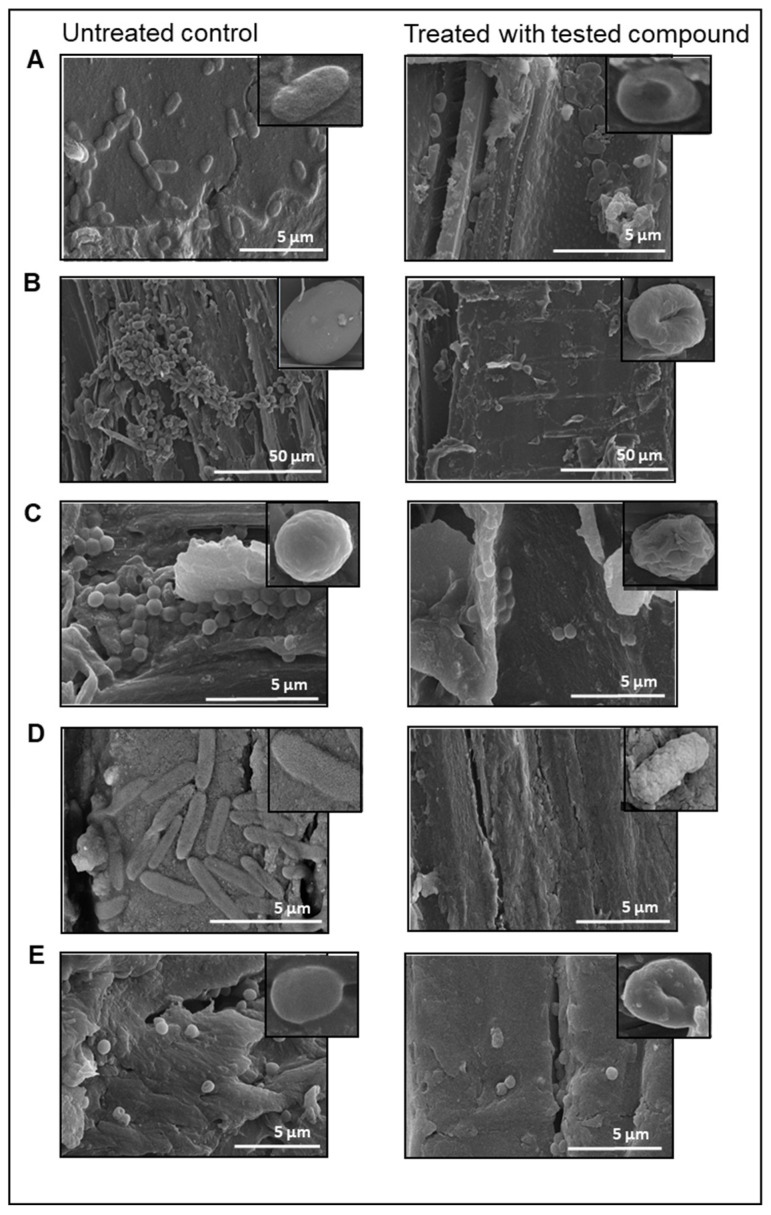
SEM images of untreated biofilms and biofilms treated with (**A**) *P. aeruginosa*, (**B**) *C. albicans*, (**C**) *S. aureus*, (**D**) *E. coli*, (**E**) *S. epidermidis*. Images represent typical fields of view.

For each pathogenic microorganism tested, the MIC value was quite high (Table 1). The highest MIC values were recorded for *S. epidermidis*, *S. aureus* and *E. coli*, with 412.88 mg/mL. As for the *P. aeruginosa*, the MIC resulted with a value of 275.25 mg/mL. In the case of *C. albicans*, this value was lower and amounted to 206.44 mg/mL. The obtained values were the basis for selecting the concentration of *Bacillus*-fermented supernatant (BFS) in the finished formulation for cosmetic use. For comparison, the extracts obtained after the fermentation of *B. amyloliquefaciens* showed, inter alia, the inhibitory effect of *S. aureus* and *S. epidermidis* with MIC values of 25.0 μg/mL and 12.5 μg/mL, respectively [32]. Other potential cosmetic raw materials obtained through the fermentation process also show antimicrobial activity. One example is *Lactobacillus*-fermented plant juices showing activity against *E. coli*, *S. aureus*, *P. aeruginosa*, group A *Streptococcus* and *C. albicans* [33]. In the next stage of this research, the influence of *Bacillus*-fermented supernatant on the formed biofilm and adhesion capacity was determined. The SEM analysis revealed reductions in biofilm for all tested microorganisms after exposure to BFS (Figure 3).

The untreated biofilm was compact and the test surface was densely covered. Under the influence of BFS, a reduction in the biofilm on the plate surface could be observed. This was also confirmed by the result obtained in the experiment with crystal violet. The biofilm reduction was on the level 67.41–93.16% and the greatest reduction was recorded for *S. aureus* (Table 1). Both the antimicrobial activity and the reduction in the biofilm may be due to the presence of levan in the *Bacillus*-fermented supernatant. Ağçeli and Cihangir found that levan has an antimicrobial and antibiofilm effect on pathogenic microorganisms [34]. Antibacterial activity of levan was evaluated against bacteria and the largest zone of inhibition was observed against *E. coli* at a concentration of 1000 μg/mL. Antibiofilm activity of levan was also evaluated, and results shown that levan concentrations inhibited biofilm formation of *P. aeruginosa* ATCC 27853, *S. aureus* ATCC29213, *Klebsiella pneumoniae* ATCC 4352, and *C. albicans* ATCC 10231 [34]. The morphology of the untreated cells showed their normal structure and smooth surface. Cells after BFS treatment exhibited a more irregular surface and visible morphological changes. Adhesion is the process by which microorganisms can stick to other cells or surfaces. Adhesion itself is a multi-stage process, but it is also the first stage in the formation of a microbial biofilm. The effect of BFS on the adhesion capacity was also observed. The reduction in adhesion to the polystyrene plaque ranged from 53.46 to 96.97% compared to untreated cells (Table 1).

### 2.3. Development of Formulations and Technologies to Obtain Shower Gels Containing Digestate Extract

Studies exploring the application of levan-rich digestate extract to body wash cosmetics were conducted on the basis of originally designed shower gel formulations. When selecting their composition, the authors relied on the available literature [35,36] and their own expertise in the field of cosmetic technology [1,4,10,12,37]. Different cosmetic formulations contained varying concentrations of the extract. The extract was added to the cosmetics in place of water as the primary ingredient of the product. A reference product used in the studies was a prototypical cosmetic formulated without the raw material being evaluated. A detailed list of ingredients used in different prototypical shower gels under study is given in Table 2.

The cosmetic formulations were prepared by carrying out the steps of the procedure outlined below. The prototypical cosmetics were produced using an MZUTL 5 homogenizing mixer from Urliński (producer: Urlinski, Warsaw, Poland). A total of 5 L of the cosmetic formulation was obtained in a single batch. Sodium coco sulfate was dissolved in water at 95 °C. Next, mixing was commenced (at 50 rpm), and the raw materials were added in the order specified in the formulation (up to and including the parfum). The ingredients were stirred until a clear homogeneous solution was obtained. In the next step, the pH was adjusted to 5.5. Afterwards, cocamidopropyl betaine was added and mixed into the formulation. The samples were set aside at room temperature for 24 h until the system was completely deaerated. The study samples exhibited full stability during the period of storage under normal sunlight conditions and at a normal temperature.

### 2.4. Safety Assessment of the Shower Gels

**Figure 4:**
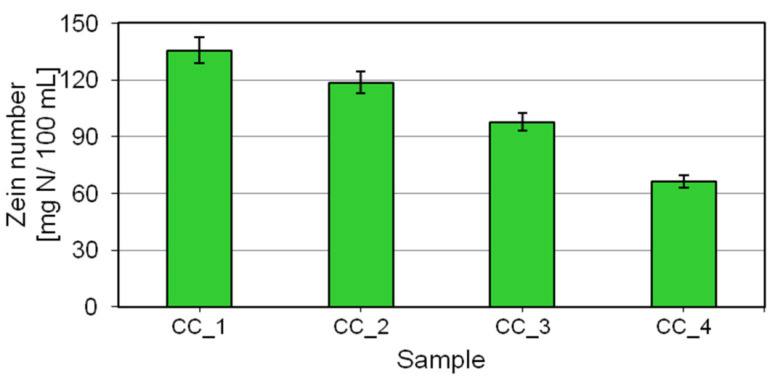
Zein value for shower gels formulated with the digestate extract.

**Figure 5:**
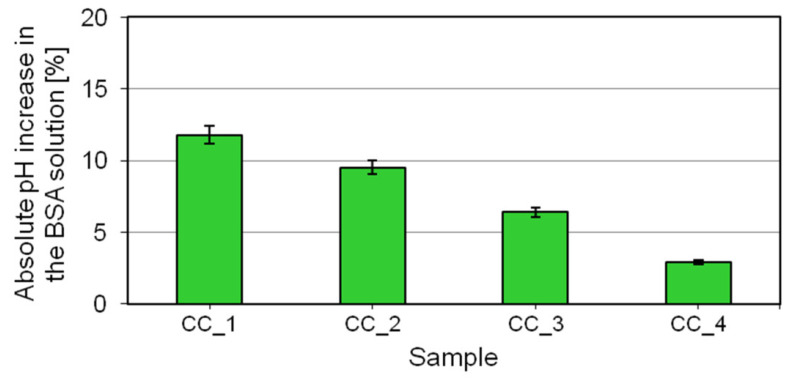
Changes in pH of the mixture of bovine serum albumin solutions and shower gels containing the digestate extract.

**Figure 6:**
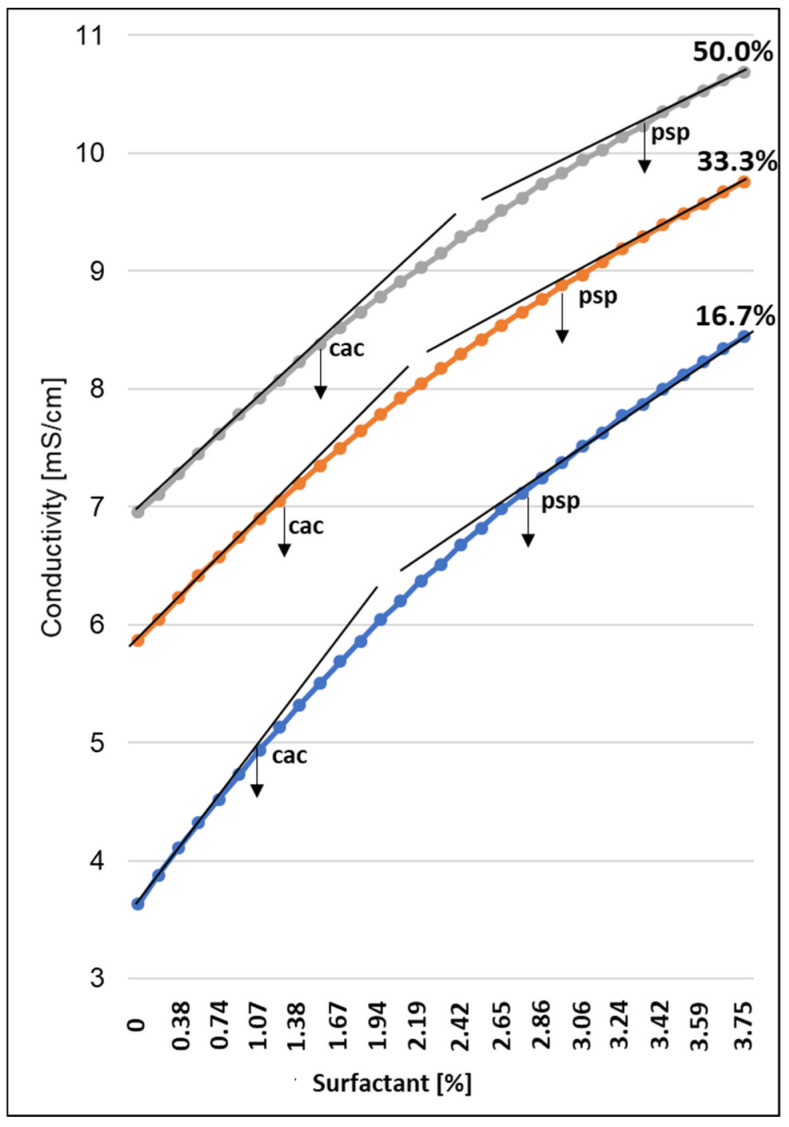
Plots of conductivity versus sodium coco sulfate concentration for various concentrations of *Bacillus*-fermented supernatant.

**Figure 7:**
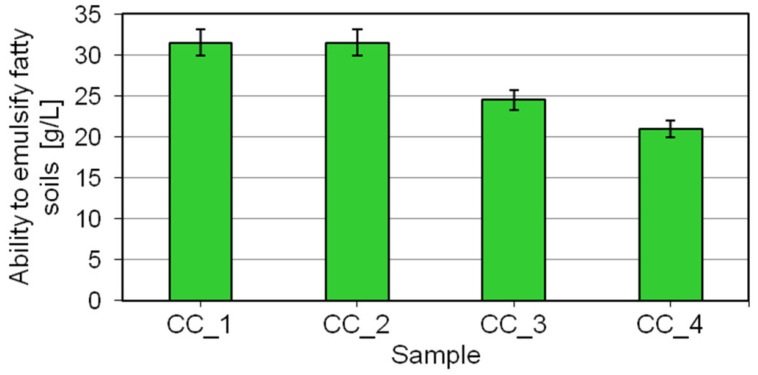
Ability to emulsify fatty soiling determined for shower gels formulated with the digestate extract.

When evaluating body wash cosmetics such as shower gels, consumers increasingly take into account the safety of using cosmetic products in terms of their potential to induce skin irritation. Consumer evaluation is particularly rigorous with regard to cosmetics marketed as “natural”. Since their production process is based on certified raw materials of natural origin, they are described as being completely environmentally friendly and safe both by consumers and, to a large extent, by manufacturers themselves. In actual fact, the safety of body wash cosmetics, understood as a limited potential to produce skin irritations, is not related to the natural origin of materials used in the formulation process. The skin irritating effect induced by body wash cosmetics depends on the type (chemical structure) and concentration of surfactants and additives used to modify product characteristics to achieve improved functional properties [9]. Tests were conducted to determine the safety of the formulated prototypical body wash cosmetics in terms of the risk of skin irritation developing after their application (Figure 4, Figure 5, Figure 6 and Figure 7).

The zein values determined for the shower gel prototypes ranged from 135 to 66 mgN/100 mL. The highest value was obtained for the formulation CC_1, containing no digestate extract. Partial replacement of water by the digestate extract in the prototypical body wash cosmetics leads to a decrease in the zein number to the minimum value in the formulation CC_4, in which the ingredient represented 50% of the composition. The zein number test (Figure 4) showed that the substitution of water by the digestate extract in the prototypical body wash cosmetics had the benefit of reducing the skin irritating effect of the entire cosmetic formulation.

The impact of the digestate extract on the skin irritating effect elicited by the body wash cosmetics containing the extract can be evaluated by performing the bovine serum albumin (BSA) test. It is based on interactions between the cosmetic’s surface-active ingredients and the water-soluble protein albumin. Anionic surfactants—used as the primary ingredients with a cleansing effect—bind to the cationic groups on the protein, causing its denaturation. In order to neutralize the negative protein charge resulting from the predominance of anionic groups in its molecules, adsorption of protons from the solvent takes place, causing a rise in the pH of the solution. The higher the pH increase compared to the baseline (5.5), the greater the skin irritating effect of the product analyzed [1]. The test results obtained for the prototypical body wash cosmetics containing the digestate extract are shown in Figure 5.

The test results (Figure 5) are consistent with the results of the zein number determination. The highest pH increase in the mixture, close to 15% compared to the baseline, was observed for the formulation CC_1. The result shows that this prototypical cosmetic was characterized by the highest skin irritating effect out of all analyzed products. Incorporating the digestate extract into cosmetic formulations results in a lower increase in pH value. The smallest pH increase in relation to the baseline was noted for the formulation CC_4, which contained the digestate extract at the highest studied concentration.

Tests evaluating the skin irritating effect of the analyzed prototypical cosmetics confirm the anti-irritation effect of the digestate extract used. The observed decrease in the skin irritating effect accompanying a rise in the concentration of the digestate extract in the cosmetics results from the composition of that ingredient, as well as from potential interactions between the chemical compounds it contains and the surfactants used as the main functional ingredients in the formulated body wash cosmetics. The effect produced by the extract is probably attributable to the significant proportion of levan in its composition (Figure 1). Furthermore, the impact of the digestate extract on reducing skin irritation may arise from its wide range of other ingredients, including proteins and mineral salts. Ingredients of this type are also capable of reducing interactions between surfactants and the skin [9].

Levan is probably the adsorption site for free surfactant monomers, which effectively reduces their concentration in the bulk phase. This restricts potential interactions involving monomers and the epidermal surface, thereby decreasing the skin irritating effect of the entire formulation. In addition, the surfactant micelles forming in the solution may bind relatively permanently with levan chains, creating a polymer–surfactant complex with a beaded structure. The stability of micelles in aggregates of this type is higher than the micelles forming in the bulk phase without the involvement of macromolecular compounds. This results in an equilibrium shift in the bulk phase towards more stable micellar aggregates and a decline in the concentration of free monomers in the solution. As a consequence, the skin irritating effect of the formulation becomes significantly reduced [9,38]. The claim is corroborated by the results of the tests shown in Figure 6.

Polymers and surfactants are very commonly included in many industrial products, and their mixtures can exhibit molecular interactions affecting the properties of the product. In this respect, the mechanism of interaction between a water-soluble polymer—levan and an anionic surfactant Sodium Coco Sulfate (SCS), occurring in greater quantity was investigated. The addition of SCS to the *Bacillus*-fermented supernatant is presented in Figure 6.

There are two break points in the relation of tested surfactant and the conductivity. The first break point is related to *critical aggregation concentration* (cac) and the second break is for saturation point of the polymer by the surfactant (psp). The interactions between surfactant and polymer solution is visible when the cac value is reached. A linear increase in conductivity was observed when SCS was added to the *Bacillus*-fermented supernatant solutions, with the same slope for all solutions tested. When the cac value is reached, the slope decreases. This may suggest absorption or the formation of polymer clusters resulting in depletion of free surfactant ions in the solution. This relationship lasts until the polymer is saturated with SCS molecules, which determines the second break point or psp. Further addition of SCS results in a linear conductivity relationship and the slope of the curve is the same for all solutions. All plots show two line areas, below the cac and above the psp. Upon reaching psp, only normal surfactant micelles are formed [39]. However, this does not exclude their formation below the psp point [40]. Once the cac of the polymer is reached, the surfactant molecules begin to associate with it to form micellar structures around each polymer molecule and they remain in equilibrium with the surfactant molecules in the solution [41]. Its further addition to the polymer-containing solution results in the formation of larger micellar structures, the mobility of the ions in the solution is reduced, revealing the visible psp point [42].

The skin irritating effect of body wash cosmetics is mainly related to the interactions between basic surfactants, i.e., the primary ingredients of the formulation, and the stratum corneum, as well as the results of these interactions. In addition to potential adverse interactions with proteins building corneocytes in the stratum corneum and deactivation of enzymes known to play a role in healthy skin function [9,11,43], scientific studies analyzing the safety of cosmetics from the viewpoint of their skin effects also address the impact of cosmetic products in the context of hydrophobic components present in the epidermis [9,44,45,46]. Specifically, an excessive ability to emulsify fats in body wash cosmetics carries the risk of removing valuable lipids from the epidermal protective layer, which are proven to contribute to maintaining appropriate hydration of the epidermis and strengthening the skin’s barrier function. Such processes can disrupt the structure of the intercellular cement between corneocytes or contribute to damaging the enzymes that produce lipids in the extracellular matrix. Consequences include an increase in trans- epidermal water loss and symptoms such as dry skin, epidermal cracking or even scaling [1,46]. Increased emulsification of fatty soiling can also lead to uncontrolled removal of naturally occurring bacterial flora from the epidermal surface, inducing a change in skin pH [1,47,48]. On the other hand, the ability of cosmetic formulations to emulsify fatty soiling in the wash bath guarantees appropriate product functionality which is expected by consumers in terms of washing performance. This is because the mechanism of skin washing consists of a number of processes aimed at removing a variety of soils from the skin surface, including environmental pollutants and skin secretions (e.g., sweat or sebum), one of the key subprocesses being emulsification of soils in the wash bath solution [1,49,50]. It ensures permanent removal of soils from the skin surface. The results obtained in the evaluation of the ability of the shower gels to emulsify fatty soiling are shown in Figure 7.

The ability of the studied prototypical cosmetics to emulsify fatty soiling was shown to be in the range of 31.5–21.0 g/L. The highest ability to emulsify fats was shown for the shower gel CC_1 which was formulated without the digestate extract. The addition of the extract leads to a considerable decrease in the parameter determined for the cosmetics. At the maximum studied concentration of the digestate extract (sample CC_4), the level of fatty soiling that could be emulsified in the wash bath was found to be the lowest. The results of the study show that the incorporation of the digestate extract into body wash cosmetics significantly impairs their functionality in terms of the ability to emulsify fats. On the other hand, a reduction in this parameter provides clear benefits, making cosmetics highly safe to use in terms of their interactions with epidermal lipids and potentially associated effects, such as excessively dry skin after product application. Similar findings were reported by Ananthapadmanabhana et al. [45,46,51], who evaluated the ability of model surfactant solutions to solubilize stearic acid and cholesterol. The authors assert that the documented limited capacity of some surfactants to remove hydrophobic components naturally occurring in the epidermis makes them safer for the skin. Researchers argue that the approach to the quality of body wash formulations in terms of their impact on the skin must be comprehensive, taking into account individual ingredients of the formulation but also looking at the product as a whole, with an emphasis on interactions with both epidermal proteins and lipids. Similar conclusions are reported in [1,2,51], with the authors pointing out that cosmetics formulated specifically for body washing purposes are expected to remove relatively loose soiling typically found on the skin. Consequently, an excessive ability to emulsify fatty soiling is not desirable in their case. The composition of body wash cosmetics should be adjusted appropriately. On the one hand, cosmetic products should have good cleansing properties. On the other, they should not cause excessive removal of lipids which are essential for healthy skin functioning.

### 2.5. Evaluation of Functional Properties

Ensuring the desirable functionality of body wash cosmetics involves appropriate selection of their quantitative and qualitative composition, so that the finished product displays satisfactory performance in the eyes of consumers. In their evaluation, consumers are concerned primarily with the characteristics related to the cosmetic’s performance (cleansing action) and application (good foaming ability, and appropriate viscosity and rheological properties). With regard to cosmetics marketed as “natural”, which are free of colorants enhancing their visual appeal, consumer evaluation also includes the aspect of color. Such cosmetics are typically transparent systems, and their color results from the raw materials used for production. The section below outlines the results of studies showing how the addition of the digestate extract affects the functionality-related aspects of cosmetic quality. The analysis comprised a range of parameters including viscosity, color, foaming ability, detergent properties and rheological characteristics of prototypical shower gels.

**Figure 8:**
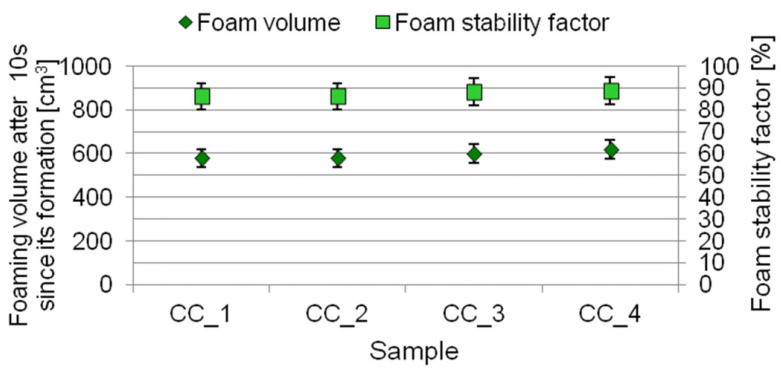
Foaming ability of shower gels containing digestate extract.

The results obtained in the assessment of foaming ability of the shower gels are shown in Figure 8.

The shower gels under study are characterized by very good foaming ability. Their aqueous solutions generate between 580 and 620 cm^3^ of foam. Foam stability in all the cosmetics under study was found to be within a similar range of 86–89%. The concentration of the digestate extract was not shown to have an effect on the properties of the aqueous solutions of the studied body wash cosmetics.

**Figure 9:**
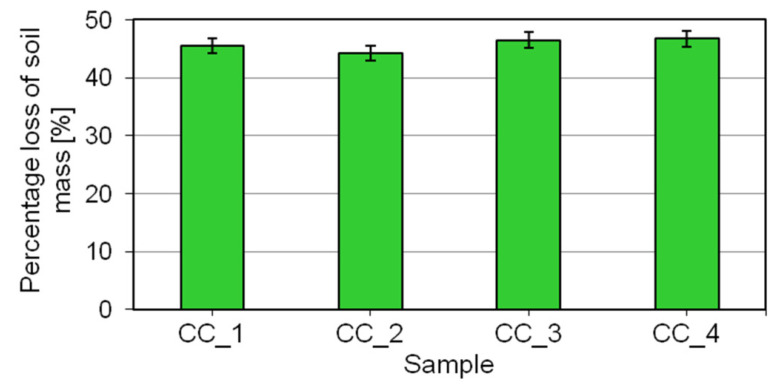
The evaluation of detergent properties for the shower gels.

The results obtained in the evaluation of detergent properties of the shower gels are shown in Figure 9.

The addition of the digestate extract has no impact on the detergent properties of the prototypical shower gels analyzed in the study. The decreases in the weight of soiling determined after the contact with the solutions of the cosmetics under study were similar and stood at around 45% of the baseline value.

Since the goal was to develop products that meet the criteria of natural cosmetics, no colorants were added to the formulations. The shower gels under study are transparent systems. Colorimetric evaluation was performed to determine how the application of the digestate extract affected the natural color of the products arising from the raw materials used. The results of the colorimetric analysis are given in Table 3.

The base shower gel was very light in color, with a shade close to yellow (h^o^ value was around 93). Preparations containing extracts from the digestate also had a very light shade, similar in shade to yellow, slightly changing to green (h^o^ range 101.9–102.6). On the basis of the determined values of ΔE_shower gel with digestate extract/base shower gel_, it was found that the use of digestate extracts slightly affects the color difference of individual shower gels. The calculated ΔE_shower gel with digestate extract/base shower gel_ values were close to the range from about 1.0 to 2.0. This proves that the color difference is only slightly discernible, only by an experienced observer [52,53].

An important parameter for evaluating the quality of shower gels is viscosity. Consumers often mistakenly believe that highly viscous body wash cosmetics are rich in active substances. As a consequence, the parameter is essentially equated with product efficacy. In fact, the viscosity of cosmetics formulated as aqueous surfactant solutions is typically adjusted by adding a salt (usually NaCl) or a polymeric viscosity modifier. The measure is applied to facilitate product application. Moreover, viscosity can affect the ease of dispensing the product from the package and spreading it over the skin, and reconstitution with water to obtain a wash bath [54,55].

**Figure 10:**
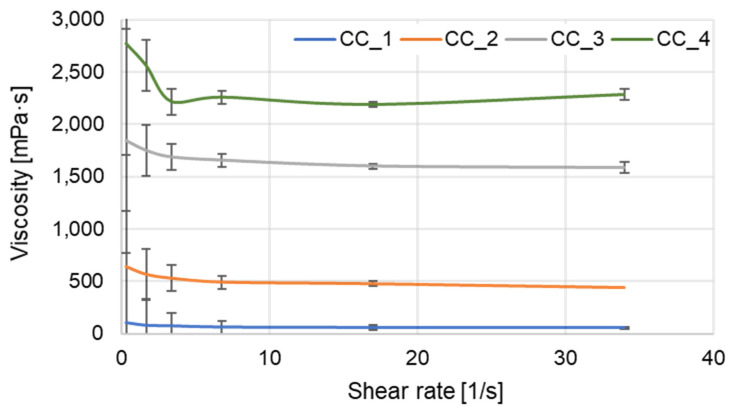
Shear rate dependence of viscosity for shower gel with digestate extracts.

Concentration-dependent viscosity curves were recorded for the investigated products. The effect of adding-fermented extract at different concentrations (17, 33 and 50%) on viscosity with increasing shear rate is shown in Figure 10.

The shower gels showed similar viscosity curve profiles, with an increase in viscosity upon addition of the fermented extract.

**Figure 11:**
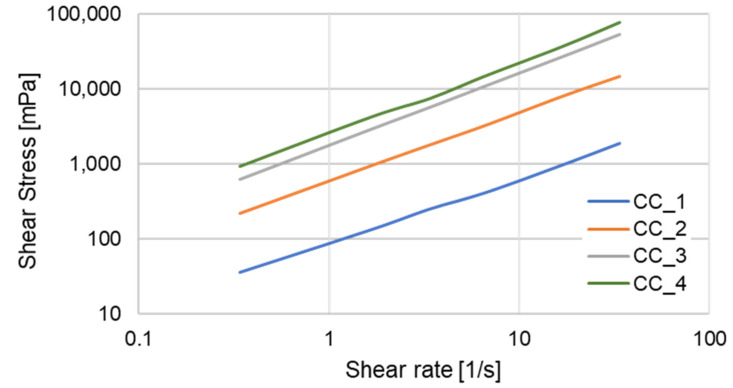
The flow behavior of shower gels a function of shear rate.

The flow behavior of shower gels was also investigated as a function of shear rate (Figure 11).

It was observed that the shear stress increased with increasing concentration and increased with the increase in shear rate. Calculation of the flow behavior index, *n*, using the power low model (Ostwald de Waele model) revealed the shear-thinning behavior of shower gel CC_1, without addition of fermented extract, with *n* < 1 characteristic for pseudo-plastic material. After addition of the fermented extract, it was observed that *n* value was close to 1, indicating the changing behavior of the shower gels towards Newtonian fluids with viscosity independent of the share rate.

Increasing the concentration of fermented extract caused increases in shower gel viscosity. The product designed without extract addition exhibited the lowest viscosity. The viscosity of the shower gel (share rate 34 s^−1^) with addition of the 17% of extract was approximately 10 times higher than that of the CC_1 product. The viscosity of the shower gels with concentrations of 33 and 50% fermented extract were about 30 times and 40 times higher, respectively.

## 3. Materials and Methods

### 3.1. Raw Materials for Cosmetics

The shower gels were made with certified raw materials of plant origin and approved for the production of natural products according to COSMOS standards: sodium coco sulfate (trade name: Sulfopon 1216G, supplier: BASF, Germany), coco glucoside (trade name: Plantacare 818, BASF, Germany), cocamidopropyl betaine (trade name: Dehyton K45, supplier: BASF, Germany), sodium benzoate and potassium sorbate as preservatives (trade name: KEM BS, supplier: Akema Fine Chemicals, Italy), *Bacillus* Ferment Extract (supplier: InventionBio, Poland), citric acid (trade name: citric acid, supplier: POCH Poland), sodium hydroxide (sodium hydroxide, supplier: POCH Poland), distilled water.

### 3.2. Fermentation Process for Levan Production with Bacillus Subtilis Natto KB1

The inoculation of *Bacillus subtilis* natto KB1 was carried out in a bioreactor with a total capacity of 5 L in LB medium (10 g/L bacto-tryptone, 5 g/L bacto-yeast extract, 10 g/L NaCl). The process was carried out for 24 h at the temperature of 37 °C with constant stirring (200 rpm) and aeration (1 vvm). The starting OD was 0.1. Fermentation was carried out in a dedicated medium (sucrose 50 g/L, MgSO_4_ × 7H_2_O − 0.5 g/L, NaH_2_PO_4_ × 2H_2_O − 3 g/L, Na_2_HPO_4_ × 12H_2_O − 3 g/L). During the process, the pH of the culture was controlled with 1 M NaOH and 1 M HCl. Foaming was controlled with almond oil. Upon completion of the process, the biomass was removed by centrifugation (17 000× *g*). The supernatant was divided into two parts and one part was freeze-dried for analytical studies and the other part was used for further application research.

### 3.3. Bacillus-Fermented Supernatant Analysis

#### 3.3.1. NMR analysis

^1^H nuclear magnetic resonance (NMR) spectra were recorded using the AVANCE III NMR 500 MHz spectrometer (Brucker Co., Billerica, MA, USA) at 25 °C. Preparation of the samples for the study included precipitation by using ethyl alcohol in a ratio of 1: 4, centrifugation of the precipitate and its lyophilization. Then, the sample was dissolved in deuterated water (D_2_O). The chemical shifts (δ) were obtained as ppm. The obtained chemical shifts were compared with the previously obtained results [16] relating to the *B. subtilis* KB1 strain used.

#### 3.3.2. Fourier-Transform Infrared Analysis

The infrared spectra were recorded with a Bruker Vertex 70 FT-IR spectrometer. The sample precipitated after fermentation and was prepared as described above, as a KBr pellet. Sample was scanned over a wavelength range of 4000–400 cm^−1^. The obtained spectrum was compared with the previous data obtained for the *B. subtilis* KB1 strain used.

#### 3.3.3. Determination of Levan Content

The levan concentration in the post-fermentation product was determined using the Fructan Assay Kit K-FRUC (Megazyme). For determinations, a specific volume of the Bacillus-fermented supernatant sample was precipitated with 4 portions of cold 96% ethanol, left overnight, and then the pellet was centrifuged and lyophilized. The levan content was determined according to manufacturer’s protocol. The method consists of enzymatic and chemical removal of sugars, i.e., sucrose, glucose and fructose, from the sample, and then of a colorimetric determination of fructane content after its hydrolysis. Absorbance was measured at 410 nm against a reagent blank. The calculations were performed by using the downloaded Megazyme Mega-Calc for Fructan Assay Kit [56]. Maleic acid, p-hydroxybenzoic acid, sodium borohydride and sodium citrate dihydrate were purchased from Sigma. Other reagents (NaOH, CH_3_COOH, calcium chloride dihydrate) were of analytical grade and purchased from PoCH (Poland).

#### 3.3.4. ICP–OES Analysis

For ICP–OES analysis, the *Bacillus*-fermented supernatant was digested in acidic conditions. The concentration of selected elements was determined by iCAP 7400 DUO emission spectrometer (Thermo Fisher Scientific) optimized and calibrated for multielement analysis taking into account the effect of the acid matrix. Samples were analyzed in triplicate.

### 3.4. Conductivity Measurements

Conductometric measurements were carried out at the temperature of 22 ± 1 °C in a beaker by adding the appropriate volume of a 4% solution of sodium coco sulfate anionic surfactant to 50 mL of solution obtained after fermentation with a concentration of 16.7, 33.3 and 50% (*v*/*v*). The surfactant was added until its concentration in the solution reached 3.3%. After adding each portion of the surfactant, the entire solution was mixed with a magnetic stirrer until a constant conductivity value was obtained. The conductance of the solution was measured on Elmeiron CPC-505 conductivity meter. All solutions were prepared with double distilled water of specific conductance between 1 and 2 µS/cm at 22 °C.

### 3.5. Antimicrobial Activity

Pseudomonas aeruginosa ATCC 9027, Staphylococcus aureus ATCC 6538, Staphylococcus epidermidis ATCC 1917, Escherichia coli ATCC 8739 and Candida albicans ATCC 10231 were used for antimicrobial tests.

#### 3.5.1. Agar Well Diffusion Test

A bacterial and yeast cell suspension obtained after overnight culture was spread uniformly on the solid agar medium and left dried at room temperature. The wells were cut using sterile Pasteur pipet and the diameter of the wells was the same in each experiment (8 mm). Then, 50 µL of *Bacillus*-fermented supernatant was loaded and kept in chilled conditions for 2 h to allow diffusion into the agar. Then, another 50 µL of tested solution was added into the wells. PBS solution was used as a negative control. Penicillin–Streptomycin and acetic acid were used as positive controls for bacteria and yeast respectively. The agar plates were incubated at 37 °C for 24 h for *S. aureus*, *P. aeruginosa*, *E. coli*, *S. epidermidis* and *C. albicans*. A clear zone diameter around the well, which indicated the microbial inhibition, was measured at two perpendicular directions. All experiments were performed with three replications.

#### 3.5.2. Minimum Inhibitory Concentration (MIC) Determination

The MIC was defined as the lowest concentration of the tested compounds at which no bacterial growth occurred. The inocula were standardized to 0.5 McFarland standard. Bacterial strains of *P. aeruginosa* ATCC 9027, *S. aureus* ATCC 6538, *S. epidermidis*, *E. coli* ATCC 8739 were grown in LB medium (BioShop) with different concentrations of Bacillus-fermented supernatant for 24 h at 37 °C in 96-well plates. As for *C. albicans* ATCC 10231, it was incubated with the same concentration of Bacillus-fermented supernatant as for the bacteria strains, for 24 h at 28 °C in YPG medium (1% YE, peptone BioShop, 2% glucose Bioshop) in 96-well plates. After the incubation period, the optical density was measured using a microplate reader at 600 nm (ASYS UVM 340 Biogenet). Negative and growth control wells did not contain tested supernatant.

#### 3.5.3. Effect of Bacillus-fermented Supernatant on Pathogen Cell Shape—Scanning Electron Microscopy (SEM)

Selected strains were incubated in 96-wells microplates with *Bacillus*-fermented supernatant for 24 h at 37 °C. After this time, the wells were washed 2 times with PBS buffer and prepared for SEM analysis. First the cells were fixed with 2.5% glutaraldehyde in PBS, then dehydrated in a series of acetone washes and dried. The SEM analysis was performed on Hitachi S-3400N equipped with a tungsten cathode (magnification 80–300.000×) at operation voltage of 15 keV at room temperature.

#### 3.5.4. Effect of Bacillus-fermented Supernatant on Pathogenic Strain Biofilm Formation

The protocol was based on our previous experiments [57]. Bacterial strains were grown on LB medium at 37 °C for 24 h and *C. albicans* ATCC 10231 was grown in YPG medium at 30 °C for 24 h. The overnight cultures were centrifuged and washed twice with PBS buffer. Then the cells suspensions were prepared with an optical density OD_600_ = 1 for bacteria strains and OD_600_ = 0.6 for *C. albicans*. Then 100 µL of each suspension was added to the wells and incubated for 2 h in 37 °C on a rotary shaker (MixMate, Eppendorf, Germany) at 300 rpm. After that time, the microbial suspension was removed and wells were washed twice with PBS buffer. Then, 100 µL of *Bacillus*-fermented supernatant was added to each well, and for the negative control, 100 µL of PBS was used. The plate was incubated for another 2 h in 37 °C on a rotary shaker at 300 rpm. Then the wells were washed twice with PBS and the cells were stained with 0.1% crystal-violet for 5 min at room temperature and washed in triplicate with PBS. Then, 150 µL of isopropanol-0.04 N HCl and 50 μL of 0.25% SDS per well was added to solubilize the dye. The absorbance of each well was measured using a microplate reader at 590 nm (ASYS UVM 340 Biogenet). The results were expressed as a percentage of control (untreated cells). Assays were carried out twice in three replications.

#### 3.5.5. Pre-adhesion Activity of Bacillus-Fermented Supernatant

Bacterial strains were grown as it is described in Section 3.4. The protocol was adapted from [58] with some modifications. The *Bacillus*-fermented supernatant was tested for its pre-adhesion activity in 96-well plates (Sarsteadt, Germany). The wells were filled with 100 µL of *Bacillus-fermented* supernatant and incubated for 2 h at 37 °C on a rotary shaker (MixMate, Eppendorf, Germany) at 300 rpm. After 2 h, the wells were washed twice with PBS. Negative control wells contained only PBS buffer. The overnight cultures of tested strains were centrifuged, washed twice with PBS and resuspended to an optical density OD_600_ = 1 for bacterial and OD_600_ = 0.6 for C. albicans. Then, 100 µL of prepared microbial suspensions were added to the wells and incubated for another 2 h at 37 °C on a rotary shaker at 300 rpm. After that, the wells were washed in triplicate to remove nonadherent cells. The adherent cells were stained with 0.1% crystal-violet for 5 min at room temperature and then the wells were washed three times with PBS. The dye was resolubilized with 150 µL of isopropanol-0.04 N HCl and 50 μL of 0.25% SDS per well. The absorbance of each well was measured using a microplate reader at 590 nm (ASYS UVM 340 Biogenet). The results were expressed as a percentage of control (untreated cells). Assays were carried out twice in three replications.

### 3.6. Zein Test

Irritant potential of the shower gel was measured using the zein test. The study was carried out using the automatic mineralization system Digestor 8AR and the automatic nitrogen analyzer Kjeltec 8400 (Producer FOSS, Denmark). In the zein test procedure, 2 g of protein was solubilized in 40 g solution of cosmetic sample (10% wt.). The amount of solubilized protein was determined by Kjeldahl analysis, and the result of the zein number procedure was expressed as mg of solubilized protein (calculated as nitrogen) in 100 mL of sample. The final result was the arithmetic mean of three independent measurements. The test methodology was described in more detail by Nizioł-Łukaszewska et al. [4] and Bujak et al. [2,12,38].

### 3.7. pH Rise Test with Bovine Albumin Serum (BSA)

The test was based on measuring the degree of protein denaturation by determining the pH level of the BSA solution in the solution of the studied cosmetic. The greater its increase, the stronger the skin irritating effect produced by the product concerned. The results were expressed as a percent increase in the pH value in relation to the level defined for normal human skin (pH = 5.5). Three independent assays were performed for each of the studied cosmetics and the results were averaged. The test methodology was described in more detail by Seweryn et al. [13] and Bujak et al. [38].

### 3.8. Evaluation of Ability to Emulsify Fatty Soils

The ability to emulsify fatty soils was evaluated in tests conforming to the PN-C-77003 standard. The maximum weight of rapeseed oil colored with Sudan Red (0.1 g of Sudan IV per 1000 mL of rapeseed oil) capable of being emulsified by 1 dm3 of a 1% aqueous solution of the studied cosmetics was determined. The experiments were carried out as follows: 1.4 g of rapeseed oil colored with Sudan Red (model fatty soil) and 2.0 g of the studied cosmetics were placed in a 50 mL beaker. Then the mixture was intensively stirred with a glass rod (diameter of the rod, 7 mm; rotational speed, about 200 rpm) for 5 min. The mixture obtained was transferred quantitatively into a 200 cm^3^ volumetric flask and brought up to volume with distilled water. The flask was closed and rotated for 5 min with rotations of 180° (one rotation per second). The resulting emulsion was placed in an incubator (45 °C) for 30 min. The flask was then taken out, and the emulsion was assessed. Separation of the oil layer in the flask’s neck or the appearance of one or more drops of colored oil in the upper part of the flask’s neck was considered to be a negative result (i.e, the liquid was not capable of emulsifying a given weight of fatty soil). When a negative result was obtained, subsequent trials were carried out in which the weight of oil was decreased by 0.2 g. If the result obtained was then positive, subsequent trials were carried out (with an increase in the oil weight of 0.2 g) until a negative result was obtained. The test consisted of determining the maximum weight of rapeseed oil which can be emulsified by 1 L of a washing bath containing 1 wt% of the evaluated formulation. The final result (mean value of three independent measurements) obtained in the test determining the ability of the evaluated formulation to emulsify fatty soils was expressed in grams of oil per liter of the evaluated formulation at the concentration of 1 wt%. The test methodology was described by Seweryn et al. [1,13].

### 3.9. Evaluation of Foaming Properties

The method of measurement was in line with Polish Standard PN—EN 1272. The experiments were carried out as follows: 100 cm^3^ of 1% aqueous solution of studied body wash cosmetic was poured into a glass cylinder. Then, the foam was whipped (time of whipping 60 s., number of full hits 60) using a perforated disc placed on a metal bar. The volume of the foam formed was read out after 10 s. Foaming ability was described as foam volume 10 s after its formation. Additionally, the percent foam stability coefficient was evaluated as a ratio of the foam volume after 10 min. The final result was the arithmetic mean of three independent measurements.

### 3.10. Evaluation of Detergent Properties

The detergent properties were evaluated based on the methodology described in the US patent No. 4904359 [59].

A 3 g portion of fatty soiling (pork lard) was applied to a pre-weighed (with an accuracy to 0.01 g) 250 mL polypropylene beaker. After being liquefied by heating, the lard was spread evenly on the bottom of the container. Next, the beaker was placed in the refrigerator for 1 h and then brought to room temperature. The aim of this step was to fix the soiling in the container. In a separate beaker, a 250 g of portion of 0.4% aqueous solution of the study formulation was prepared and the temperature set at 46 °C. After the solution had reached the desired temperature, it was poured into the beaker containing the soiling. The beaker was placed in the incubator for 15 min and the temperature set at 46 °C to keep the temperature of the solution constant. After 15 min, the beaker was emptied, rinsed gently with distilled water using a wash bottle, and dried for 1 h in a drying oven at 46 °C to completely evaporate the solution. Following that time, the beaker was weighed again. By comparing the difference in weight between the beaker containing 3 g of lard and the empty beaker, the decrease in weight of the soiling was calculated. The final result was expressed as a percentage loss of the weight of the fatty soiling following contact with the aqueous solution of the studied formulation.

### 3.11. Rheological Properties

The viscosity was measured at 20 °C using a Brookfield rheometer DV2TRV with Small Sample Adapter and Cylindrical Spindle SC4 (Brookfield, St. Louis, MI, USA). For each test, 8 mL of the sample was used. Different shear rates and shear stresses were applied to the sample, and the resulting rheogram was constructed to determine the rheological behavior. All measurements were carried out in triplicate.

### 3.12. Determination of the Color Parameters

Samples of cosmetics with digestate extracts were tested at room temperature, 48 h after their preparation. A CHROMA METER CR-400 (Konica Minolta, Sensing Inc., Japan) was used to evaluate the color parameters (CIELAB coordinates). The CIELAB system was defined by the International Commission on Illumination in 1978. It is based on three color attributes: L*, a*, b*, where L* is a brightness variable proportional to the value in the Munsell system, and a* and b* are chromatic coordinates. The a* and b* coordinates indicate positions on the red/green and yellow/blue axes, respectively (+a = red, −a = green; + b = yellow, −b = blue).

Based on the data obtained: L*, a* and b*, the following color parameters were calculated: chroma (C*) and hue (h^o^). The following equations were used:

(1) $$C^{*}=\sqrt{{\left(a^{*}\right)}^{2}+{\left(b^{*}\right)}^{2}}$$

(2) $$h^{o}=\arctan\frac{b^{*}}{a^{*}}$$

General color difference (ΔE_shower gel with digestate extract/base shower gel_) was calculated according to the following formula:

(3) $$\Delta E_{\mathrm{shower gel with digestate extract/base shower gel}}^{*}=\sqrt{{\left(\Delta L^{*}\right)}^{2}+{\left(\Delta a^{*}\right)}^{2}+{\left(\Delta b^{*}\right)}^{2}}$$

where: ΔL*, Δa*, and Δb* are the mathematical differences between shower gel with extracts L*, a*, b* and base shower gel L*, a*, b* values.

## 4. Conclusions

The study showed that the levan-rich digestate extract was a suitable ingredient for the formulation of body wash cosmetics which are safe on the skin. The prototypical shower gels prepared for this purpose were subjected to tests evaluating their skin irritation potential and basic parameters related to functionality. The zein numbers determined for the cosmetics containing the highest analyzed concentration of the digestate extract were over 50% lower compared to the water-based reference formulation. Consistent results were obtained in the BSA test, indicating a significant reduction in skin irritation characteristics after the application of the studied ingredient to the formulation. The effect is attributed to the high content of protein and mineral salts in the extract and predominantly to the presence of levan. As shown by conductometric studies, this polymer, through its impact on anionic surfactants, successfully prevents interactions between these compounds and epidermal structural proteins, thus reducing the skin irritating effect. Evaluation of the ability to emulsify fatty soiling showed a significant decrease in the evaluated parameter accompanying an increase in the concentration of the digestate extract. A decrease in the value of this parameter may translate into reduced impact of the formulation on epidermal lipids, and thus into the improved safety of the cosmetics regarding their effect on the skin. Other studies evaluating functionality indicated that the application of the digestate extract did not affect the functional performance of the shower gels under study. Rheological analysis revealed a thickening effect of the extract, which may be due to the presence of considerable amounts of electrolytes in the composition of this raw material. Consequently, there is no need to incorporate an additional viscosity modifier into the formulation to obtain the desired viscosity level and application parameters expected by consumers.

## Figures and Tables

**Table 1:** Influence of *Bacillus*-fermented supernatant on selected pathogenic strains—inhibition zone, MIC value, biofilm formation and adhesion to the polystyrene surface.

Pathogen	Inhibition Zone [mm]	MIC Value [mg/mL]	Biofilm * [%]	Adhesion * [%]
*P. aeruginosa* ATCC 9027	4.50 ± 1.38	275.25	92.75	53.46
*S. aureus* ATCC 6538	2.83 ± 0.68	412.88	67.41	68.02
*S. epidermidis* ATCC 1917	3.42 ± 0.49	412.88	92.12	72.42
*E. coli* ATCC 8739	2.50 ± 0.84	412.88	93.16	96.97
*C. albicans* ATCC 10231	5.17 ± 0.75	206.44	76.83	70.36

* Values expressed as a percentage of untreated cells. Experiments performed in triplicate.

**Table 2:** Model shower gel formulations containing digestate extract.

Name According to INCI ^1^	CC_1	CC_2	CC_3	CC_4
Aqua	to 100			
Sodium Coco Sulfate	3.3			
Coco Glucoside	4.2			
*Bacillus* Ferment Extract	0	16.7	33.3	50.0
Cocamidopropyl Betaine	1.5			
Citric Acid/ Sodium Hydroxide	do pH 5.5			
Sodium Benzoate, Potassium Sorbate	0.45			
Parfume	0.5			
Aqua	to 100			

^1^ INCI = International Nomenclature of Cosmetic Ingredients.

**Table 3:** Color parameters for cosmetic (shower gel).

	L*	a*	b*	C*	h^o^	ΔE_shower gel with digestate extract_ _/base shower gel_
CC_1	92.3	−0.29	5.47	5.5	93.0	-
CC_2	93.1	−0.93	4.41	4.5	101.9	1.47
CC_3	94.0	−0.96	4.31	4.4	102.6	2.16
CC_4	93.5	−1.00	4.76	4.9	101.9	1.56

## Data Availability

Data are contained within the manuscript.
